# Posterior Interosseous Nerve Schwannoma Presenting as a Painless Forearm Mass: A Case Report

**DOI:** 10.7759/cureus.103815

**Published:** 2026-02-18

**Authors:** Radhika G Nair, Manal M Khan, Ved Prakash Rao Cheruvu, Prateek Jain, Arun A J

**Affiliations:** 1 Plastic and Reconstructive Surgery, All India Institute of Medical Sciences, Bhopal, Bhopal, IND; 2 Radiation Oncology, All India Institute of Medical Sciences, Bhopal, Bhopal, IND

**Keywords:** antoni a areas, antoni b areas, peripheral nerve tumor, posterior interosseous nerve, schwannoma, tinels sign, verocay bodies

## Abstract

Schwannomas are benign peripheral nerve sheath tumors that typically exhibit slow growth and may present diagnostic challenges when arising from uncommon sites. We report the case of a 65-year-old female who presented with a six-year history of a gradually enlarging, painless swelling over the extensor aspect of the left forearm. The lesion initially appeared as a small nodule and progressively enlarged to approximately 4 × 3 cm, with intermittent tingling and occasional numbness but no significant functional impairment. Clinical examination revealed a firm, well-defined mass with a positive Tinel’s sign. Ultrasonography and contrast-enhanced MRI demonstrated a well-circumscribed, encapsulated lesion along the course of the posterior interosseous nerve (PIN) in the intermuscular plane, suggestive of a benign peripheral nerve sheath tumor.

The patient underwent planned microsurgical excision under general anesthesia. Intraoperatively, the tumor was found to arise eccentrically from the PIN and was meticulously dissected from the surrounding nerve fascicles, allowing complete excision while preserving nerve continuity. Histopathological examination confirmed the diagnosis of schwannoma, demonstrating characteristic Antoni A and Antoni B areas, Verocay bodies, and thick-walled hyalinized vessels. The postoperative course was uneventful, with preservation of motor and sensory function and no evidence of recurrence on follow-up. This report underscores the importance of thorough clinical evaluation and appropriate imaging in the diagnosis of peripheral nerve tumors of the forearm and highlights the role of meticulous microsurgical technique in achieving complete tumor excision with excellent functional outcomes.

## Introduction

Schwannomas are benign peripheral nerve sheath tumors that typically present as solitary, encapsulated subcutaneous lesions in otherwise healthy individuals. Although usually isolated, they may occasionally be multiple or arise from various locations along the peripheral nervous system, including cranial nerves, spinal roots, and major nerve plexuses such as the brachial and lumbosacral regions [1]. Representing approximately 5% of all benign soft tissue tumors, schwannomas most commonly occur in the head, neck, and extremities [2]. While slow-growing and often asymptomatic, they can produce sensory disturbances, such as tingling or numbness, when adjacent nerve fibers are compressed [3]. The pathogenesis is generally attributed to sporadic somatic mutations, though associations with hereditary conditions, such as neurofibromatosis, have been reported [4].

When evaluating palpable masses of the upper limb, the possibility of a peripheral nerve tumor should be considered. Clinical features such as a positive Tinel's sign and localized paraesthesia raise suspicion for a nerve sheath lesion [5]. Imaging is essential in assessment, with MRI often demonstrating characteristic findings, including a well-defined capsule, the split fat sign, and heterogeneous T2-weighted signal intensities that aid in differentiating schwannomas from other soft tissue masses [6,7].

Although schwannomas of the posterior interosseous nerve (PIN) are uncommon, their deep location and proximity to motor branches pose significant diagnostic and surgical challenges. As noted in previous reports, successful management relies on careful preoperative planning and precise microsurgical techniques to achieve complete tumor excision while preserving nerve integrity [8-10].

## Case presentation

A 65-year-old female with a past medical history significant only for hypertension presented with a gradually progressive swelling over the left forearm that had been present for six years. The swelling had begun insidiously as a marble-sized nodule and slowly increased to approximately the size of a lemon. There was no history of trauma, pain, ulceration, discharge, or skin changes. The patient reported intermittent tingling and occasional numbness localized to the lesion, but her daily activities were not impacted. She had no other comorbidities, allergies, or relevant family history.

**Figure 1:**
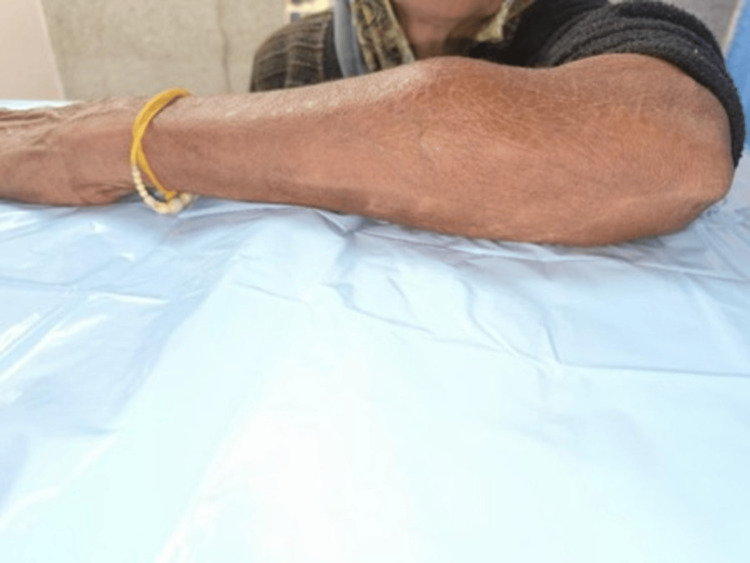
Preoperative clinical image showing forearm swelling

On examination, a solitary, well-defined, oval swelling measuring approximately 4 × 3 cm was observed over the extensor aspect of the proximal forearm. The surface was smooth with well-defined margins. The overlying skin was normal, with no erythema, ulceration, dilated veins, scar, or sinus. On palpation, the swelling was firm, non-tender, and non-fluctuant, with no local rise in temperature. It was mobile horizontally but restricted vertically, not fixed to the skin (Figure 1). It was more prominent on extensor muscle contraction, suggesting involvement of the underlying muscle plane. There was no distal neurovascular deficit. A nerve conduction study was not performed as the patient had no clinical evidence of motor deficit or denervation. Given the absence of functional impairment and the clear radiological delineation of the lesion, electrodiagnostic evaluation was unlikely to alter management. No overlying skin discoloration, warmth, or tenderness was noted. A positive Tinel's sign was present over the swelling. Motor and sensory functions of the left upper limb were normal, as were the range of motion of the elbow, wrist, and fingers. Distal pulses were palpable and symmetric.

**Figure 2:**
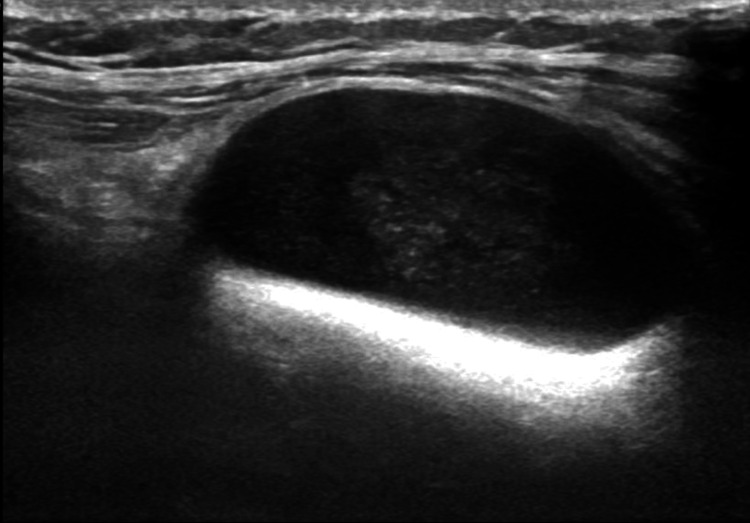
Well-defined oval-shaped hypoechoic lesion with a spindle appearance along the course of PIN PIN: posterior interosseous nerve

**Figure 3:**
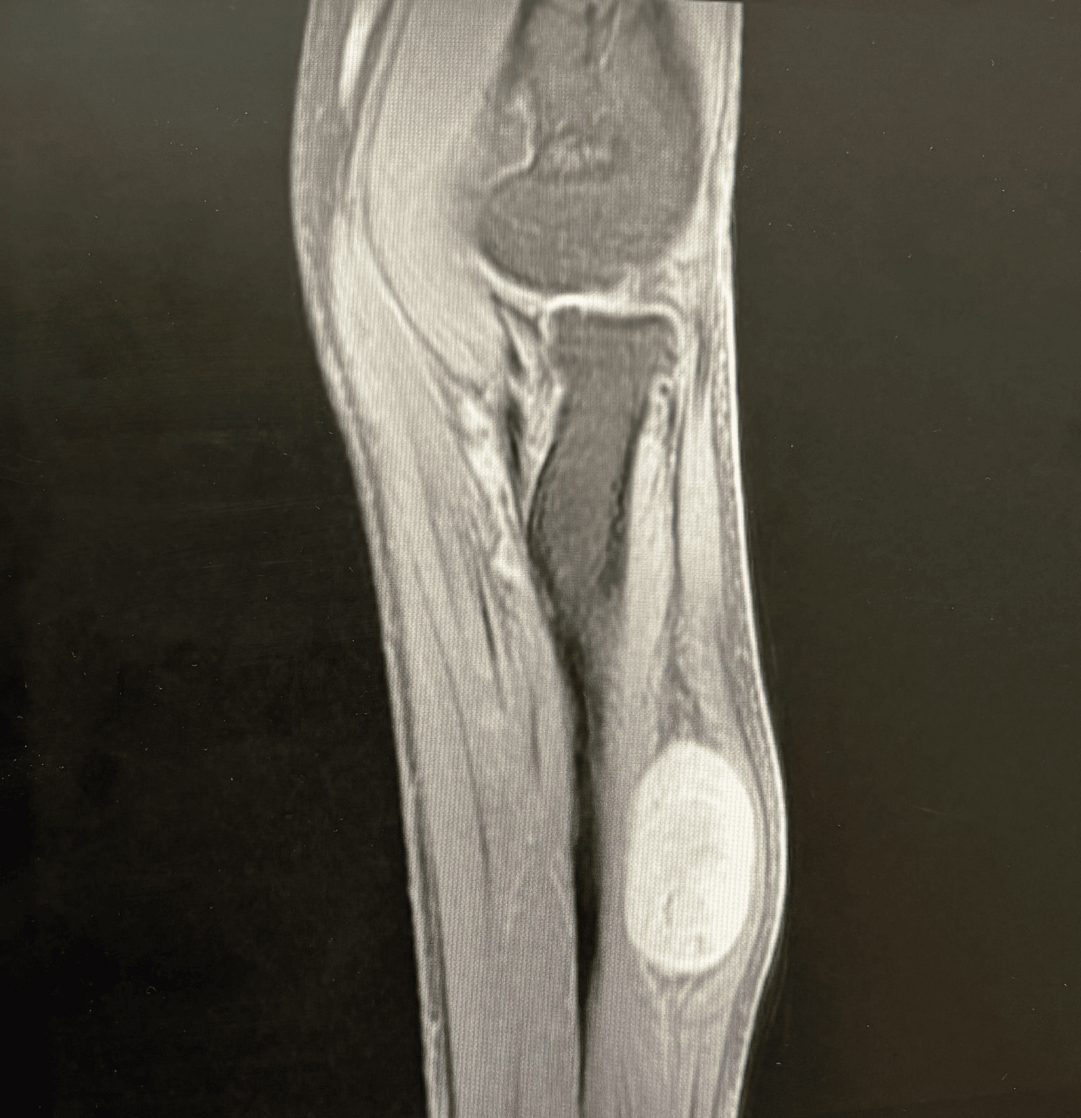
MRI sagital section showing well-defined PIN schwannoma MRI: magnetic resonance imaging; PIN: posterior interosseous nerve

**Figure 4:**
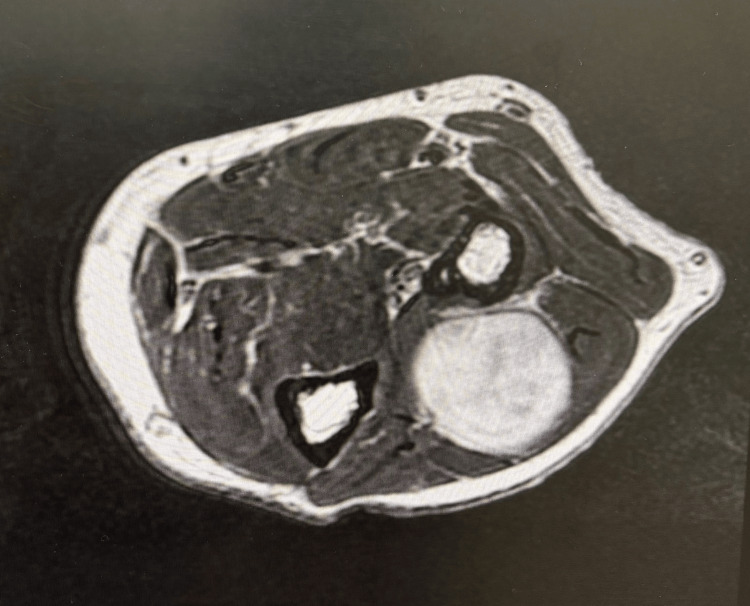
MRI axial section showing well-defined PIN schwannoma MRI: magnetic resonance imaging; PIN: posterior interosseous nerve

Initial ultrasound performed externally suggested a neuroma in the intermuscular plane. Ultrasound over the extensor aspect of the proximal forearm suggested a well-defined hypoechoic lesion with a spindle appearance along the course of PIN (Figure 2). MRI of the left forearm demonstrated a well-defined, encapsulated, oblong lesion measuring 1.7 × 1.9 × 3.2 cm located in the proximal one-third of the posterior compartment (Figures 3, 4). The lesion appeared mildly hyperintense to isointense on T1-weighted images and heterogeneously hyperintense on T2-weighted sequences, with preserved fat around its poles, consistent with the split fat sign. Multiple low-signal areas and a few blooming foci suggested internal degenerative changes. The lesion showed bright diffusion-weighted signal without corresponding ADC drop and demonstrated heterogeneous enhancement on postcontrast imaging. It abutted the abductor pollicis longus anteriorly and the extensor carpi ulnaris and extensor digitorum posterolaterally and lay along the course of the PIN. No intramuscular infiltration was observed. These findings favored a benign peripheral nerve sheath tumor arising from the PIN.

**Figure 5:**
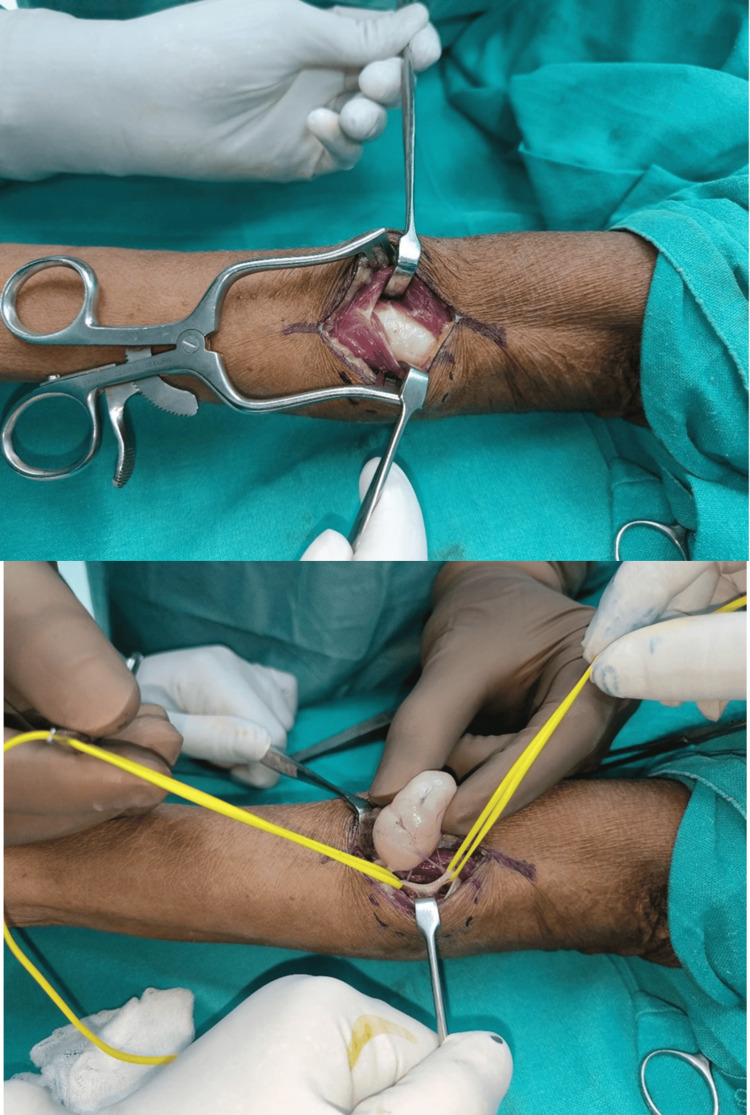
Intraoperative image - 1 An incision was made, and the extensor muscles were exposed; an encapsulated, eccentric schwannoma was identified involving the looped PIN PIN: posterior interosseous nerve

**Figure 6:**
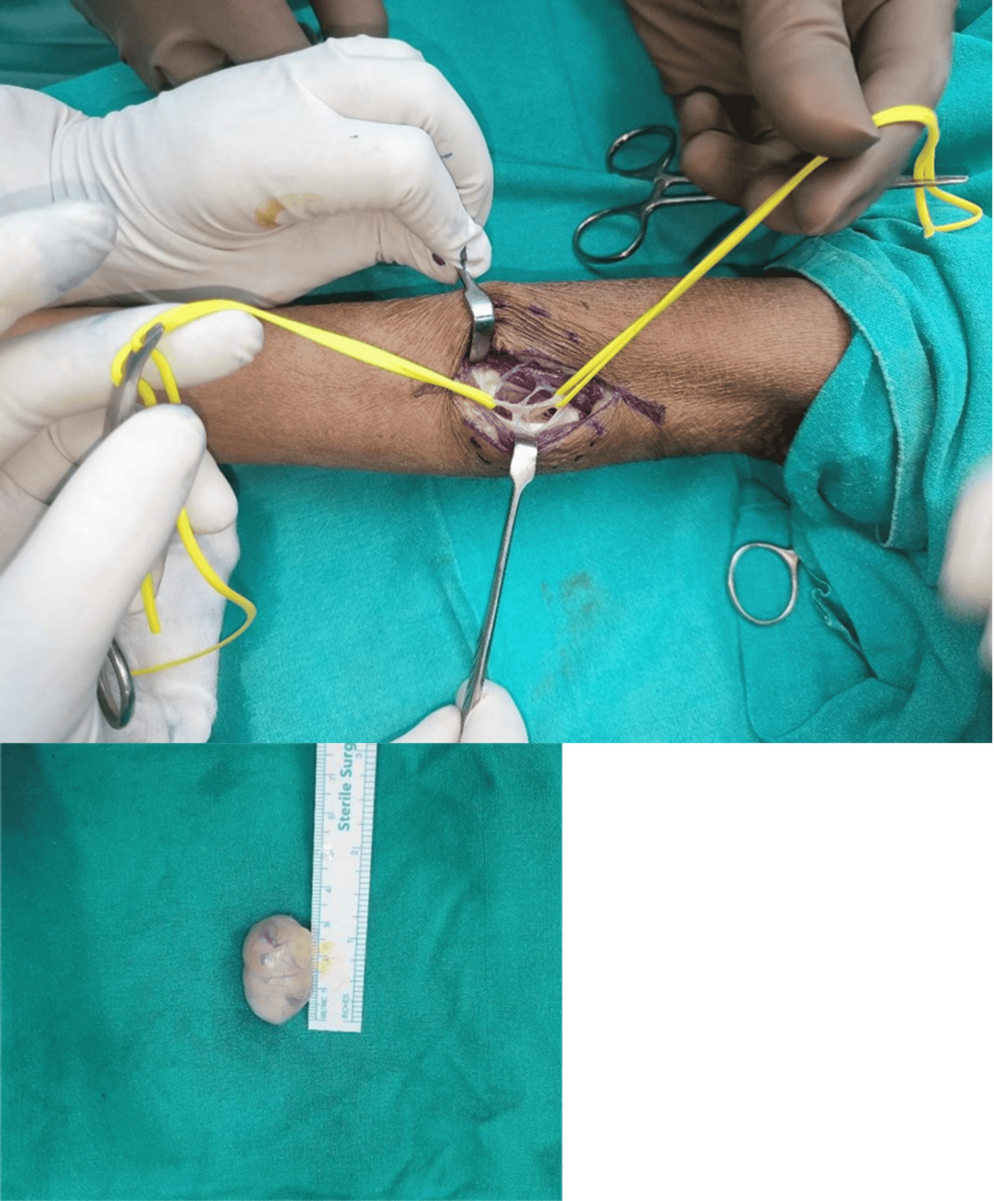
Intraoperative image - 2 Excision of the schwannoma was performed, preserving the intact PIN; the excised mass was well encapsulated and globular PIN: posterior interosseous nerve

Based on clinical and imaging features, a diagnosis of PIN schwannoma was made. The patient was admitted to the plastic surgery ward and underwent planned tumor excision under general anesthesia on January 22, 2025. Intraoperatively, a 3.5 × 1.5 cm encapsulated mass was found deep to the extensor carpi radialis longus tendon. The extensor muscles were carefully separated to expose the lesion (Figure 5). The mass was noted to arise eccentrically from the posterior interosseous nerve, with identifiable proximal and distal nerve fascicles looping around the tumor. Meticulous microsurgical dissection enabled the complete excision of the tumor while preserving the continuity and integrity of the nerve (Figure 6). The excised mass was well encapsulated and globular (Figure 6).

**Figure 7:**
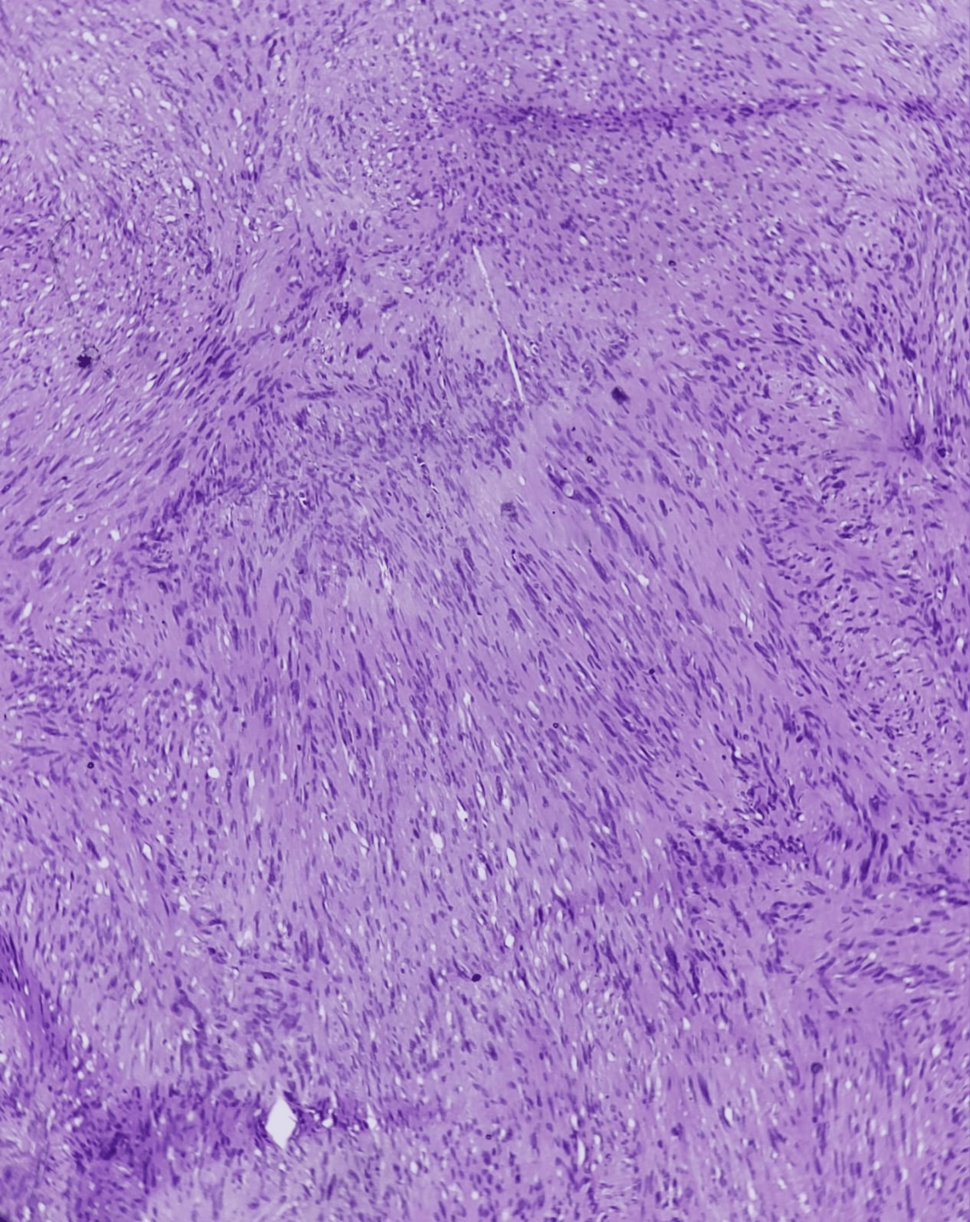
Histopathological image - Antoni A areas showing Verocay bodies

Histopathological examination revealed a capsulated gray-white mass measuring 2.4 × 2 × 1.8 cm (Figure 7). The cut surface was solid, tan, and glistening without hemorrhage or necrosis. Microscopy showed alternating Antoni A and Antoni B areas, with nuclear palisading, Verocay bodies, and thick-walled hyalinized vessels, consistent with schwannoma. No necrosis or atypical mitoses were observed.

**Figure 8:**
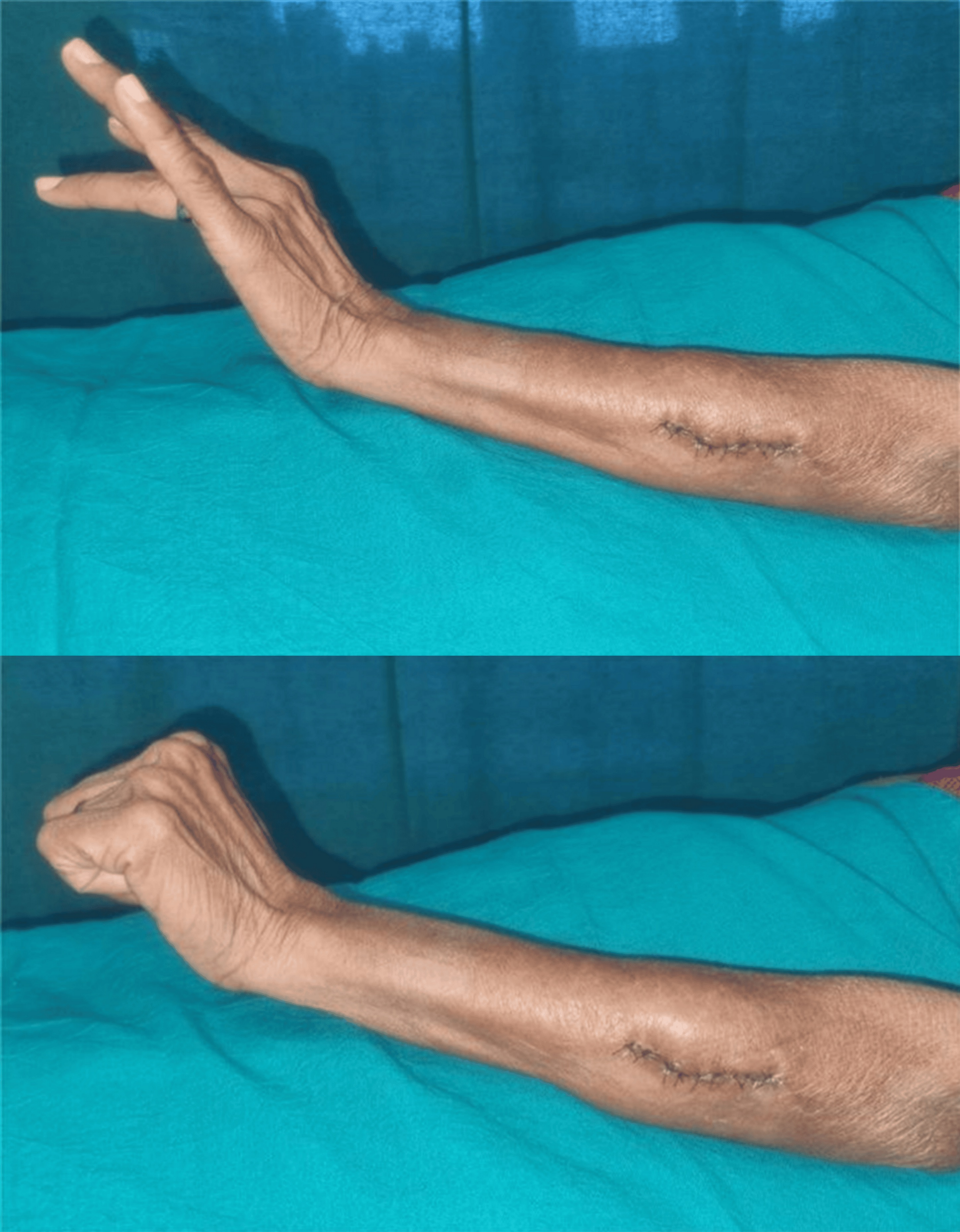
Postoperative image with sutured incision

The postoperative period was uneventful. The patient received analgesics, antibiotics, and anti-inflammatory medications. The incision site remained healthy, and there was no wound dehiscence or discharge (Figure 8). The patient showed no new neurological deficits and was discharged in stable condition.

## Discussion

This report highlights the typically indolent clinical course of schwannomas. The patient presented with a six-year history of a gradually enlarging forearm swelling, accompanied only by intermittent tingling and numbness. Such slow growth and minimal symptoms are consistent with the natural history of benign peripheral nerve sheath tumors [2,3]. The positive Tinel's sign further supported the neurogenic origin of the lesion. The imaging findings were characteristic of a schwannoma. Ultrasonography often reveals a well-defined mass with an eccentric position along the parent nerve while maintaining overall nerve continuity, a key distinguishing feature of schwannomas [8]. In this case, MRI demonstrated a well-encapsulated lesion along the PIN with features such as the split fat sign and heterogeneous T2 hyperintensity. These findings, combined with heterogeneous post-contrast enhancement, strongly supported the diagnosis of a benign neurogenic tumor [1,4,5]. The absence of intramuscular infiltration or involvement of adjacent structures reinforced the likelihood of a benign process.

Histopathological evaluation confirmed the diagnosis. The presence of alternating Antoni A and Antoni B areas, Verocay bodies, and thick-walled hyalinized vessels is typical for schwannomas. Collagen IV and reticulin staining highlighted pericellular patterns, while epithelial membrane antigen (EMA) positivity was confined to peripheral perineurial cells, findings that differentiate schwannoma from neurofibroma [9]. The lack of necrosis, atypia, and mitotic activity confirmed its benign nature.

Surgical management of schwannomas arising from the PIN poses unique challenges due to the proximity of critical motor branches. However, schwannomas are typically well-circumscribed and encapsulated, allowing careful separation from adjacent nerve fascicles. In this case, microsurgical excision achieved complete tumor removal while preserving the continuity and function of the nerve. This outcome aligns with current evidence suggesting that meticulous microsurgical dissection is the treatment of choice for peripheral nerve schwannomas [3,5]. The patient recovered without any neurological deficits, further emphasizing the efficacy of nerve-sparing techniques.

Although benign, long-standing schwannomas have the potential to cause progressive nerve compression, leading to sensory or motor deficits if intervention is delayed [2,4]. Early diagnosis and surgical treatment can therefore prevent long-term morbidity. The significance of this case is amplified by the rarity of schwannomas arising from the PIN. To our knowledge, only one similar case has been described in the literature, in which surgical removal was performed due to associated nerve palsy [7]. This report contributes to the limited body of literature on PIN schwannomas and underscores the importance of correlating clinical findings with imaging features to ensure timely and effective management.

## Conclusions

This report illustrates the typical presentation and favorable surgical outcome of a PIN schwannoma. The patient’s long-standing, painless swelling with intermittent sensory symptoms, combined with characteristic MRI findings, supported the diagnosis of a benign peripheral nerve sheath tumor. Complete excision was achieved using meticulous microsurgical technique while preserving nerve continuity and function. The uneventful postoperative recovery emphasizes the importance of timely evaluation and careful operative planning in the management of peripheral nerve schwannomas, particularly those located near critical motor branches. Early recognition and appropriate intervention can prevent long-term neurological deficits and contribute to excellent functional outcomes.
